# Imparting CO_2_ reduction selectivity to ZnGa_2_O_4_ photocatalysts by crystallization from hetero nano assembly of amorphous-like metal hydroxides†

**DOI:** 10.1039/d0ra00710b

**Published:** 2020-02-25

**Authors:** Masanori Takemoto, Yasuaki Tokudome, Soichi Kikkawa, Kentaro Teramura, Tsunehiro Tanaka, Kenji Okada, Hidenobu Murata, Atsushi Nakahira, Masahide Takahashi

**Affiliations:** Department of Materials Science, Osaka Prefecture University 1-1, Gakuencyo, Naka-ku Sakai Osaka 599-8531 Japan tokudome@mtr.osakafu-u.ac.jp; Department of Molecular Engineering, Kyoto University Kyotodaigaku Katsura, Nishikyo-ku Kyoto 615-8510 Japan

## Abstract

Imparting an enhanced CO_2_ reduction selectivity to ZnGa_2_O_4_ photocatalysts has been demonstrated by controlled crystallization from interdispersed nanoparticles of zinc and gallium hydroxides. The hydroxide precursor in which Zn(ii) and Ga(iii) are homogeneously interdispersed was prepared through an epoxide-driven sol–gel reaction. ZnGa_2_O_4_ obtained by a heat-treatment exhibits a higher surface basicity and an enhanced affinity for CO_2_ molecules than previously-reported standard ZnGa_2_O_4_. The enhanced affinity for CO_2_ molecules of the resultant ZnGa_2_O_4_ leads to highly-selective CO evolution in CO_2_ photo-reduction with H_2_O reductants. The present scheme is promising to achieve desirable surface chemistry on metal oxide photocatalysts.

## Abstract 2

The hetero-nanocomposite of Zn(OH)_2_ and Ga(OH)_3_ NPs is crystallized to ZnGa_2_O_4_ with CO_2_ affinity, showing highly-selective reaction toward CO_2_ photo-reduction.

## Graphical abstract

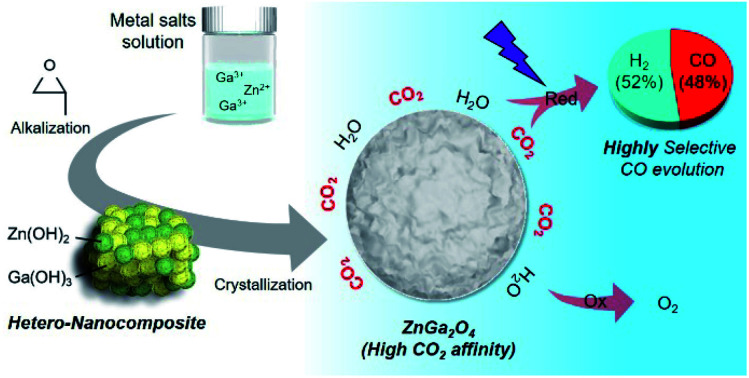

## Introduction

Heterogeneous catalysis for environmental-friendly production of resources has attracted remarkable attention.^1^ Especially, complex oxides, with high chemical/thermal stabilities and capability of their use in aqueous media, have been studied for various catalytic reactions such as CO_2_ reduction,^2–4^ water-splitting,^5,6^ biodiesel synthesis.^7^ Among many approaches, such as using co-catalysts,^8–11^ co-solvents,^12^ nanostructuration^13–16^ and Z-scheme photosynthesis,^17,18^ surface modification of catalysts is especially a promising and versatile pathway to explore attracting catalytic activities, because the adsorption of reactants on a solid surface is a key reaction step in heterogeneous catalysis.^19^

Recently, we have reported the synthesis of metal hydroxide nanoparticle (NP) with a size of single nanometer by epoxide-mediated alkalization.^20,21^ This synthesis pathway allows us to obtain metal hydroxide NPs as a stable suspension at a high yield without impurities.^22^ Moreover, a unique and tailorable surface property has been demonstrated by the introduction of a large hetero-interface by interdispersing two different hydroxide NPs in a homogenous manner.^23^ For example, hetero nanointerfaces constructed between cobalt and nickel hydroxide NPs (∼2 nm) exhibit lower charge transfer resistance and improved electrochemical properties thanks to highly homogenous interdispersion of hydroxide NPs. Our motivation in the present work is to develop a straightforward route to access a “complex oxide” photocatalyst with a desirable surface chemistry of stoichiometric composition, through a low-temperature thermal treatment on this class of metal hydroxides with hetero nanostructures.

We here choose ZnGa_2_O_4_ as a representative system in order to demonstrate the present concept for yielding metal oxide photocatalysts with an enhanced catalytic activity originated from a high surface basicity. ZnGa_2_O_4_ has been reported as a promising oxide to photocatalytically reduce CO_2_ into CO with H_2_O as an electron donor by loading of Ag co-catalyst.^24^ On the other hand, there still remains a challenge that competitive H_2_ evolution as a result of the reduction of H_2_O solvent inevitably occurs on bare ZnGa_2_O_4_ catalyst (without co-catalyst), resulting in a low reaction selectivity toward CO_2_ reduction (<∼30%).^24^ A previous report on Zn-modified Ga_2_O_3_ photocatalyst suggests that Zn–O–Ga linkages on the surface of catalysts play an important role to inhibit H_2_ production in photocatalytic conversion of CO_2_ with H_2_O.^4^ The homogenous interdispersion of precursory metal hydroxide NPs is expected to allow for the formation of such kind of surface.

Herein, we demonstrate that a nanocomposite of precursory metal hydroxide NPs with hetero nanointerfaces can be used to prepare ZnGa_2_O_4_ photocatalyst stoichiometrically-containing Zn(ii) and Ga(iii) on the oxide surface, leading to a highly-enhanced selectivity toward photocatalytic CO_2_ reduction. The precursory nanocomposite is prepared under a highly supersaturated condition induced by epoxide-mediated alkalization.^25^ ZnGa_2_O_4_ is obtained from the precursory nanocomposite by heat-treatment ranging from 500 °C to 900 °C. Surface analyses confirm that ZnGa_2_O_4_ prepared in the present scheme exhibits a higher base strength and a higher affinity for CO_2_ compared to those of standard ZnGa_2_O_4_ obtained by a solid phase reaction from a mixture of metal oxide precursors. XPS measurement has revealed that ZnGa_2_O_4_ prepared through the present scheme has a surface of stoichiometric composition that is not the case for standard ZnGa_2_O_4_ catalysts prepared at 850 °C. As a result, the present ZnGa_2_O_4_ heat-treated at 700 °C (without co-catalyst) exhibits a reaction selectivity of 48.0% toward CO evolution, more than double to that of standard ZnGa_2_O_4_ (20.3%). Also, Ag co-catalyst, that is required to maximize the reaction selectivity toward CO evolution, can be decreased into 5% of the previous reports, thanks to the high affinity for CO_2_ adsorption of the resultant ZnGa_2_O_4_ surface. Based on these insights obtained in this representative ZnGa_2_O_4_ system, synthesis of complex oxide catalysts through the crystallization of interdispersed hydroxides NPs is expected to be applied to design a wide range of complex oxide catalysts with an intended surface.

## Experimental section

### Materials

Zn(NO_3_)_2_·6H_2_O (99.0%), Ga(NO_3_)_3_·*n*H_2_O (99.9%), ZnO (99.0%), Ga_2_O_3_ (99.99%), NH_3_ aqueous solution (25 wt%), ethanol (99.5%), methanol (99.8%), bromothymol blue, phenolphthalein, 2,4-dinitroaniline, NaHCO_3_ (99.5–100.3%), 0.1 M of AgNO_3_ aqueous solution and propylene oxide (PO, >99%) were used as received. PO was purchased from Sigma-Aldrich Corp. All other reagents were purchased from FUJIFILM Wako Pure Chemical Industries. Ultra-pure water of resistivity of 18.2 MΩ cm was used in all experiments.

### Preparation of ZnGa_2_O_4_

Zn(NO_3_)_2_·6H_2_O (0.740 g; 2.49 mmol) and Ga(NO_3_)_3_·*n*H_2_O (1.99 g; 4.98 mmol, *n* is set as 8 for the calculation.) were dissolved in a mixture of ultra-pure water (6.00 mL; 333 mmol) and ethanol (6.00 mL; 165 mmol). At 25 °C, PO (8.10 mL; 112 mmol) was added to the mixture solution and the solution was stirred for 30 s to form a homogeneous sol. Then, the sol kept in a closed container was placed under a static condition at 25 °C to form a wet gel. The wet gel was aged for 24 h at 25 °C. After aging process, the wet gel was dried at 40 °C to obtain a precursory xerogel. The xerogel was calcined at various temperatures ranging from 500 °C to 900 °C for 12 h to yield ZnGa_2_O_4_. ZnGa_2_O_4_ was also prepared by a solid state reaction. The details are described in ESI.†

### Photocatalytic conversion of CO_2_ with H_2_O reductant in liquid phase

Photocatalytic conversion of CO_2_, including the step of Ag-loading on ZnGa_2_O_4_, was carried out in a flow system using an inner-irradiation-type reaction vessel at 35 °C. Photocatalyst (0.5 g) was dispersed in ultra-pure water (1.0 L) containing 0.1 M of NaHCO_3_. Ag co-catalyst was loaded on ZnGa_2_O_4_ by photo-deposition method as follows; (1) AgNO_3_ was added at a concentration ranging from 2.34 × 10^−6^ M to 46.8 × 10^−6^ M. (2) Ar gas was bubbled into the suspension at a flow rate of 80 mL min^−1^ to purge air for 1 h. (3) The flow rate was changed to 30 mL min^−1^ and the suspension was illuminated with a 400 W high-pressure mercury lamp for 2 h. These steps for Ag-loading were skipped when photocatalytic reaction over the bare ZnGa_2_O_4_ photocatalyst was examined. Then, CO_2_ gas was bubbled into the suspension at a flow rate of 60 mL min^−1^ for 1 h in order to purge Ar or air. The suspension was illuminated for 5 h with a 400 W high-pressure mercury lamp with a quartz filter equipped with a water cooling system, with a CO_2_ flow at a rate of 30 mL min^−1^. The outlet of the reactor was connected to a six-way valve with a sampling loop. Evolved gaseous products of H_2_, O_2_, and CO together with CO_2_ gas were collected and analyzed by thermal conductivity detector-gas chromatography (TCD-GC) using a GC-8A chromatograph (Shimadzu Corp.) with a Molecular Sieve 5A column and Ar as a carrier gas, and by flame ionization detector-gas chromatography (FID-GC) with a methanizer (Shimadzu Corp.), a Shin Carbon ST column, and N_2_ as a carrier gas. In an isotopic experiment with ^13^CO_2_, the formation of ^13^CO was detected by mass spectroscopy by using a quadrupole-type mass spectrometer (BEL Japan, BEL Mass).

### Characterization

Crystal structures of samples were identified by powder X-ray diffraction (PXRD; MultiFlex, Rigaku, Japan). X-ray photoelectron spectroscopy (XPS) spectra were recorded by using X-ray photoelectron spectrometer (ESCA-3400, Shimadzu Corp.) under Mg Kα radiation. X-ray absorption fine structure (XAFS) spectra of Zn K-edge and Ga K-edge were collected in the transmission mode at room temperature on BL11 at the SAGA Light Source (Saga, Japan). A silicon (111) double-crystal monochrometor was used to obtain the incident X-ray beam. The intensity of the incident and transmitted X-ray was monitored by an ionization chamber. N_2_ adsorption–desorption isotherms at −196 °C were obtained on a volumetric gas adsorption apparatus (BELSORP-mini II, Bel Japan Inc., Japan). Prior to the measurements, sample powders were pretreated at 200 °C under a vacuum condition overnight. Specific surface area of ZnGa_2_O_4_ was estimated by the Brunauer–Emmett–Teller (BET) method. Morphologies of Zn–Ga AMH and ZnGa_2_O_4_ catalysts were observed by a field emission scanning electron microscope (FE-SEM; S-4800, Hitachi, Japan, with a thin Pt coating), transmission electron microscopes (TEM; JEM-2000FX, JEOL, Japan and Hitachi 7650, Hitachi, Japan) and a field emission transmission electron microscope (FE-TEM; JEM-2100F, JEOL, Japan) equipped with EDS. The UV-Vis diffusion reflectance spectra of ZnGa_2_O_4_ samples were measured by using UV-Visible spectrometer (V-670, JASCO, Japan) equipped with an integrating sphere. The energy gap (*E*_g_) was estimated with the Davis–Mott method.^26^ Base strength of bare ZnGa_2_O_4_ catalysts was estimated by Hammett indicators; bromothymol Blue (p*K*_a_ = 7.2), phenolphthalein (p*K*_a_ = 9.8) and 2,4-dinitroaniline (p*K*_a_ = 15.0) were used as coloring agents, and methanol was used as a solvent. When the color change of the indicator is observed, the base strength (*H*_) of the tested solid sample is determined to be higher than the p*K*_a_ value of the indicator employed.^27,28^ The affinity between CO_2_ and the surface of ZnGa_2_O_4_ catalysts was assessed by thermal programmed desorption (TPD: BELCAT-B, Bel Japan Inc., Japan) of CO_2_. He gas was flowed as a carrier gas and ZnGa_2_O_4_ was degassed at 200 °C for 1 h. Then, CO_2_ was introduced and allowed to adsorb on ZnGa_2_O_4_ at 30 °C for 30 min. After the adsorption of CO_2_, the sample was heated from 30 °C to 800 °C at a heating rate of 5 °C min^−1^. Desorbed CO_2_ was monitored by using a quadrupole-type mass spectrometer (BEL Mass, Bel Japan Inc., Japan).

## Results and discussion

### Synthesis of ZnGa_2_O_4_ through calcination of a gel composed of interdispersed metal hydroxide NPs with low crystallinities

**Fig. 1:**
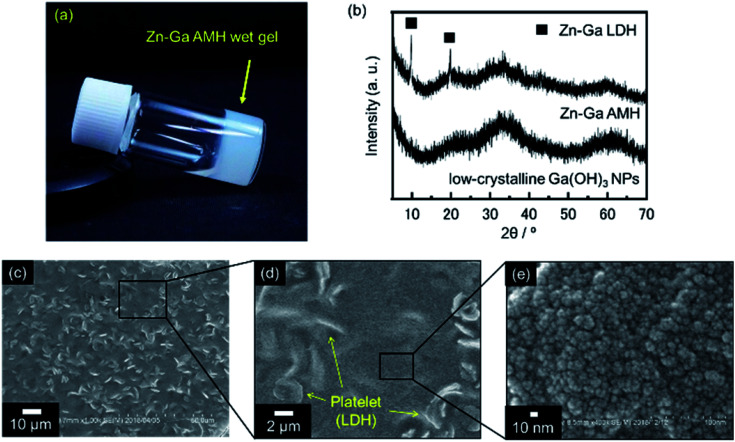
(a) Photograph of precursory wet gel obtained from an aqueous solution of Zn(NO_3_)_2_·6H_2_O and Ga(NO_3_)_3_·*n*H_2_O under a highly supersaturated condition induced by epoxide-mediated alkalization. (b) PXRD patterns of precursory xerogel and low-crystalline Ga(OH)_3_ NPs obtained from an aqueous solution of Ga(NO_3_)_3_·*n*H_2_O (without Zn(NO_3_)_2_·6H_2_O). (c–e) SEM images of precursory xerogel at different magnifications.

A precursory xerogel for the synthesis of ZnGa_2_O_4_ was obtained through epoxide-mediated alkalization from an aqueous solution of Zn(NO_3_)_2_·6H_2_O and Ga(NO_3_)_3_·*n*H_2_O (Fig. 1(a)). Two small peaks at 2*θ* = 9.9° and 19.8° and the broad diffraction pattern were observed in PXRD pattern of precursory xerogel (Fig. 1(b)). Two small peaks are assigned to 003 and 006 diffraction peaks of Zn–Ga layered double hydroxide (LDH) crystal,^29^ whose Zn^2+^/Ga^3+^ ratio was estimated as 1.5 from TEM-EDS mapping shown in Fig. S1.† By Bragg's law using the diffraction peak at 2*θ* = 9.9°, the interlayer separation is calculated to be 0.89 nm, which corresponds to an interlayer space of LDH accommodating nitrate anions.^30,31^ The broad diffraction on the PXRD pattern of the precursory xerogel is in good agreement with that for low-crystalline Ga(OH)_3_ NPs with a size of 4.4 nm in diameter (*S*_BET_ = 349 m^2^ g^−1^). The obtained gel is a nanocomposite of poorly-crystallized (amorphous-like) metal hydroxides containing a small amount of Zn–Ga LDH. The present epoxide-mediated alkalization allows us to yield a hydroxide gel corresponding with starting chemical composition because most of Zn^2+^ and Ga^3+^ species (96.3% of initial metals) are consumed during the reaction in a homogeneous manner (Fig. S2†). Scanning electron microscope (SEM) observation reveals that LDH platelets with a lateral size of 2 μm are embedded in the gel matrix composed of NPs (<10 nm in diameter) (Fig. 1(c–e)). Zn and Ga are homogeneously distributed in the precursory gel (Fig. S1(c)†), which confirms that both amorphous-like Zn(OH)_2_ and Ga(OH)_3_ NPs interdispersedly assembles to form the precursory gel (Fig. 1(e)). The present synthesis condition allows us to achieve a high degree of supersaturation and thereby the formation of abundant NPs to form a homogenous gel throughout the reaction solution. Hereafter, the present precursory composite gel is called as “Zn–Ga AMH” (Zn–Ga Amorphous Metal Hydroxide).

**Fig. 2:**
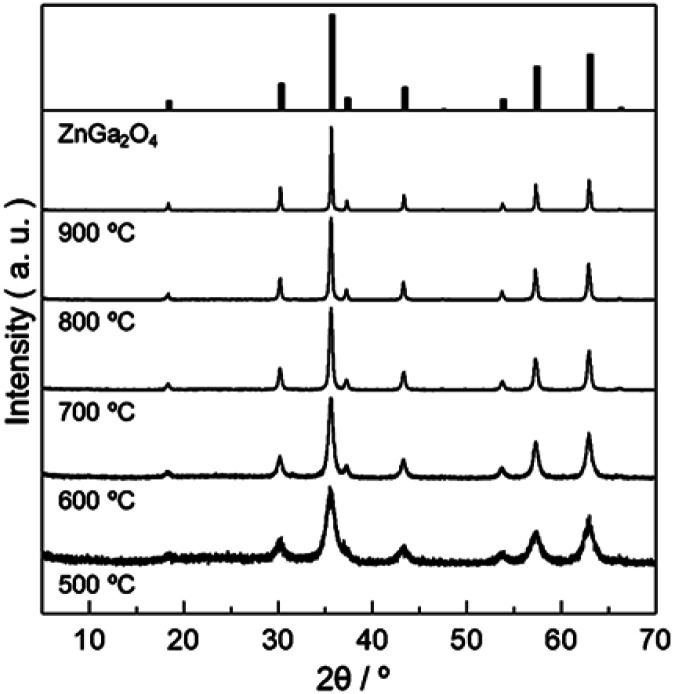
PXRD patterns of ZnGa_2_O_4_ obtained from Zn–Ga AMH by calcination in air at various temperatures for 12 h.

Fig. 2 shows powder X-ray diffraction (PXRD) patterns obtained from Zn–Ga AMH by the calcination at various temperatures ranging from 500 °C to 900 °C. Their diffraction patterns are ascribed to the typical pattern of ZnGa_2_O_4_ (ICDD PDF 00-038-1240). Interestingly, the synthesis of ZnGa_2_O_4_ as a single phase is possible even when the calcination temperature is as low as 500 °C by using the present Zn–Ga AMH as a precursor. Morphological observation by SEM (Fig. S3†) reveals that the size of ZnGa_2_O_4_ crystals obtained from Zn–Ga AMH by the calcination at 500 °C is >10 nm and becomes larger with increasing calcination temperature due to crystal growth. The achievement of low-temperature synthesis of ZnGa_2_O_4_ as a single phase is mainly due to highly-homogeneous dispersion of low-crystalline Zn(OH)_2_ and Ga(OH)_3_ NPs in Zn–Ga AMH as discussed in the former paragraph. As is well-known, cations are homogeneously distributed in hydroxide layers of Zn–Ga LDH in atomic scale,^32,33^ suggesting that the contaminated small amount of Zn–Ga LDH also contributes to the low-temperature synthesis of ZnGa_2_O_4_. ZnGa_2_O_4_ samples obtained as a single phase from Zn–Ga AMH are hereinafter named as “AMH-derived ZnGa_2_O_4_-*X*” (*X* is calcination temperature).

### Surface basicity and affinity for CO_2_ of ZnGa_2_O_4_

**Fig. 3:**
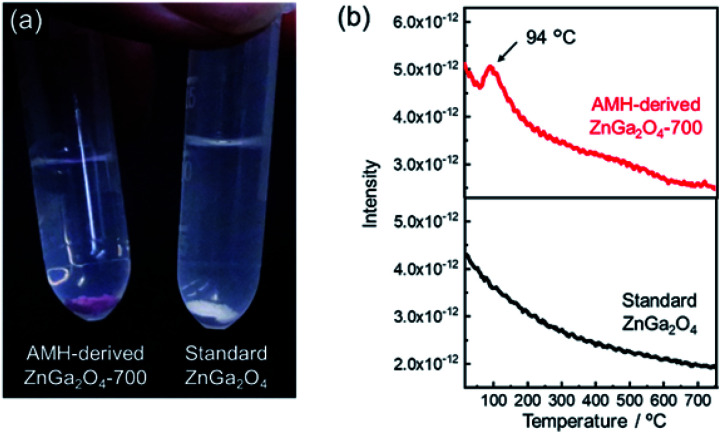
(a) Photograph of AMH-derived ZnGa_2_O_4_-700 and standard ZnGa_2_O_4_ immersed in a methanolic solution of Hammett indicator (phenolphthalein). (b) CO_2_-TPD curves of AMH-derived ZnGa_2_O_4_-700 and standard ZnGa_2_O_4_ (sample mass: 0.5 g).

The surface basicity of AMH-derived ZnGa_2_O_4_ was investigated in comparison with previously-reported one. Hereafter, ZnGa_2_O_4_ prepared at 850 °C through a solid phase reaction (Fig. S4†), whose catalytic activity is reportedly highest in the report^24^ is called as “standard ZnGa_2_O_4_”. We found that ZnGa_2_O_4_ obtained from Zn–Ga AMH exhibits a unique surface nature different from standard ZnGa_2_O_4_. Fig. 3(a) shows AMH-derived ZnGa_2_O_4_-700 and standard ZnGa_2_O_4_ immersed in a methanolic solution of Hammett indicator (phenolphthalein). AMH-derived ZnGa_2_O_4_-700 changes its color from white to red upon the immersion in the indicator solution, whilst the change of color is not observed for standard ZnGa_2_O_4_, indicating that base strength of AMH-derived ZnGa_2_O_4_-700 in methanol is stronger than that of standard ZnGa_2_O_4_. Base strength, crystallite size, and specific surface area of ZnGa_2_O_4_ samples are summarized in Table S1.† Base strength of AMH-derived ZnGa_2_O_4_ decreases by the heat treatment above at 700 °C. Nonetheless, the base strength of AMH-derived ZnGa_2_O_4_-900 is found to be still higher than that of standard ZnGa_2_O_4_ prepared at a relatively lower calcination temperature of 850 °C. The difference in coloring behavior cannot be explained by difference in surface area, but difference in surface nature; AMH-derived ZnGa_2_O_4_-900 (*S*_BET_ = 0.53 m^2^ g^−1^) exhibits a stronger surface basicity than standard ZnGa_2_O_4_ (*S*_BET_ = 1.3 m^2^ g^−1^). The epoxide-mediated alkalization employed in the present study clearly contributes to yield ZnGa_2_O_4_ with the high basic strength.

CO_2_ temperature programmed desorption (CO_2_-TPD) curves of AMH-derived ZnGa_2_O_4_-700 and standard ZnGa_2_O_4_ are plotted in Fig. 3(b). The peak at 94 °C, originated from desorption of CO_2_, is observed only for the TPD curve of AMH-derived ZnGa_2_O_4_-700, suggesting that the surface of AMH-derived ZnGa_2_O_4_ has a higher affinity and a stronger adsorptive nature for CO_2_ than standard ZnGa_2_O_4_. The peak at 94 °C could be detected even when the sample mass of AMH-derived ZnGa_2_O_4_-700 was decreased to 1/5 in order to compensate the difference in *S*_BET_ between AMH-derived ZnGa_2_O_4_-700 (*S*_BET_ = 5.0 m^2^ g^−1^) and standard ZnGa_2_O_4_ (*S*_BET_ = 1.3 m^2^ g^−1^). The result reveals that the difference in *S*_BET_ is not the cause of the different CO_2_ adsorption capacity of the two samples. The peak in low-temperature range is also observed for TPD curves of AMH-derived ZnGa_2_O_4_-500 and AMH-derived ZnGa_2_O_4_-600 (not shown). From the above all results, we can conclude that single phase ZnGa_2_O_4_ with a high base strength and a high affinity for CO_2_ can be obtained by the calcination of Zn–Ga AMH.

**Fig. 4:**
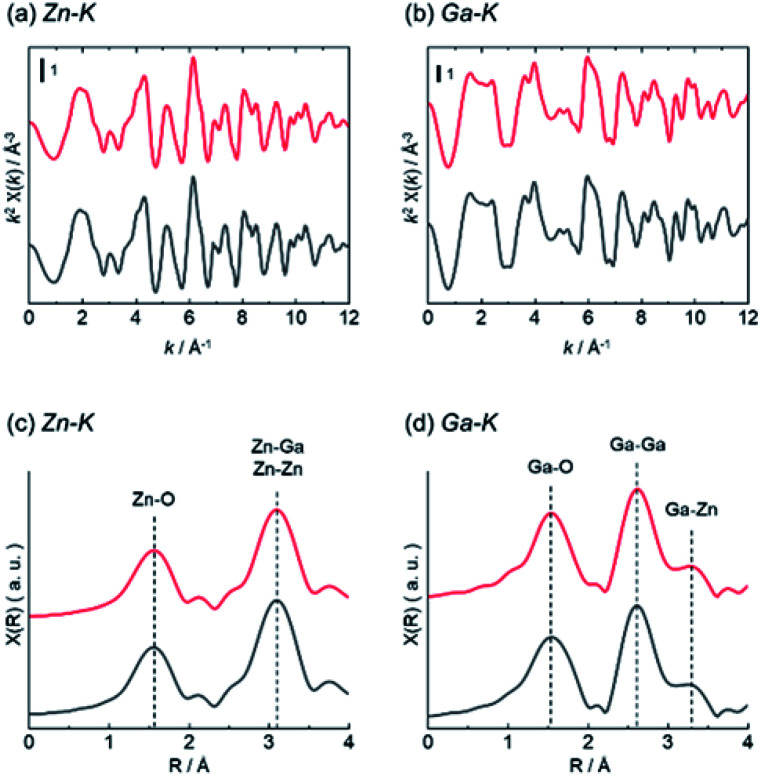
(a) Zn K-edge and (b) Ga K-edge EXAFS (c and d) Fourier transforms of EXAFS spectra of AMH-derived ZnGa_2_O_4_-700 (red line) and standard ZnGa_2_O_4_ (black line).

Structure analysis of ZnGa_2_O_4_ catalysts were performed by XAFS and XPS. The difference of surface structure between AMH-derived ZnGa_2_O_4_ and standard ZnGa_2_O_4_ was investigated by XPS. XAFS measurement was employed as a technique to understand local environments of the atoms for bulk samples. Fig. 4 shows Zn K-edge and Ga K-edge EXAFS and Fourier transforms (FT) of EXAFS spectra of AMH-derived ZnGa_2_O_4_-700 and standard ZnGa_2_O_4_. The same characteristic distance is observed in the first neighbor peaks (Zn–O shells in Zn K-edge EXAFS and Ga–O shells in Ga K-edge EXAFS) and the second neighbor peaks (Zn–Ga and Zn–Zn shells in Zn K-edge EXAFS, and Ga–Ga and Ga–Zn shells in Ga K-edge EXAFS). These results support that the average local environments of Zn and Ga atoms are comparable in AMH-derived ZnGa_2_O_4_-700 and standard ZnGa_2_O_4_.

**Fig. 5:**
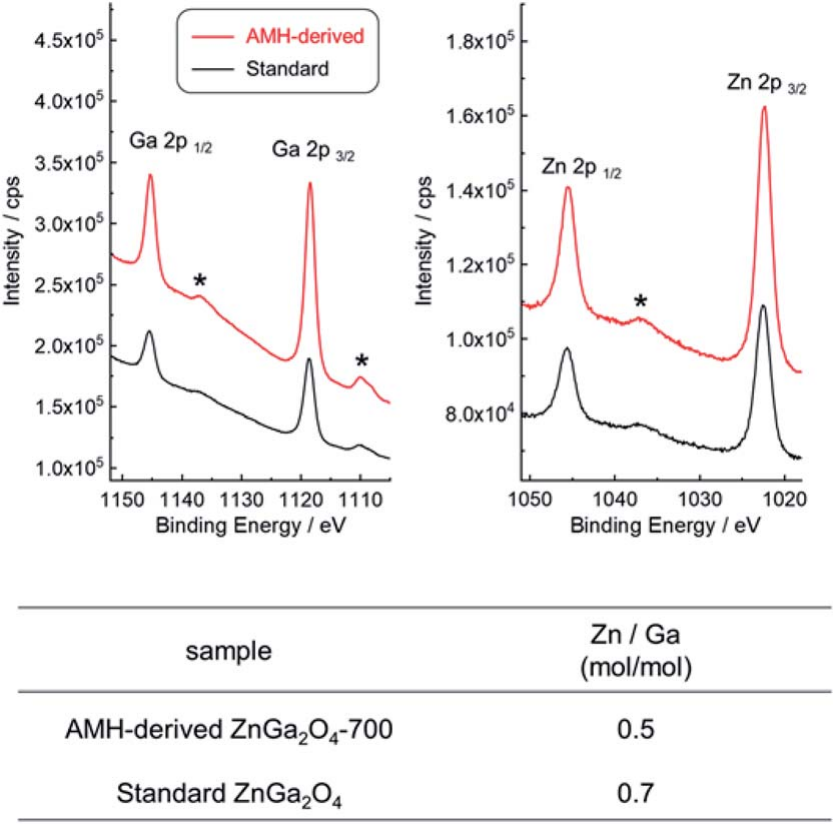
Narrow scan XPS spectra of AMH-derived ZnGa_2_O_4_-700 (red line) and standard ZnGa_2_O_4_ (black line) and the values of molar ratio of Zn/Ga. Surface chemical composition of ZnGa_2_O_4_ photocatalysts analyzed by XPS measurement *: satellite peaks.

XPS analysis was employed to compare the chemical composition for AMH-derived ZnGa_2_O_4_-700 and standard ZnGa_2_O_4_, where the sampling depth is estimated to be *ca.* 3.0 nm. The XPS peaks of Zn 3d, Ga 3d, Zn 3p, Ga 3p, Zn 3s, Ga 3s, O 1s, Zn 2p and Ga 2p are observed in wide-scan XPS spectra of both ZnGa_2_O_4_ catalysts (Fig. S5†). Narrow spectra and estimated molar ratio values of Zn/Ga of two ZnGa_2_O_4_ catalysts are summarized in Fig. 5. The peak positions of Zn 2p and Ga 2p in narrow scan XPS spectra are comparable for two ZnGa_2_O_4_ catalysts. It should be highlighted that there is a clear difference in Zn/Ga ratio between two ZnGa_2_O_4_ photocatalysts. Preferential segregation of Zn on the surface (Zn/Ga = 0.7) is confirmed for standard ZnGa_2_O_4_. A higher temperature would be required to obtain ZnGa_2_O_4_ with the surface of stoichiometric composition in this solid state reaction, whereas the calcination at a higher temperature promotes the sintering of complex oxide particles, leading to decrease of accessible sites by reactants on complex oxide catalyst. On the other hand, the stoichiometric Zn/Ga molar ratio (Zn/Ga = 0.5) on the surface is confirmed for AMH-derived ZnGa_2_O_4_-700, thanks to the highly-homogeneous distribution of hydroxide NPs in Zn–Ga AMH precursor. The results of XPS analyses suggest that a larger amount of Zn–O–Ga linkages is formed in the case of AMH-derived ZnGa_2_O_4_-700 compared to standard ZnGa_2_O_4_. Although the further study is required to elucidate exact adsorption sites on the surface for CO_2_, such as hydroxyl groups, and oxygen in Zn–O–Ga linkages, it can be concluded that AMH-derived ZnGa_2_O_4_-700 with the stoichiometric surface exhibits the higher affinity for CO_2_ molecules because of the introduction of the stronger Lewis basic sites. The use of interdispersed amorphous Zn and Ga metal hydroxide NPs is a key to yield complex oxide with the surface of stoichiometric composition even by a low-temperature synthesis.

### Photocatalytic CO_2_ reduction over bare ZnGa_2_O_4_ (without co-catalyst)

Photocatalytic properties of AMH-derived ZnGa_2_O_4_ catalysts were systematically investigated under the experimental condition optimized in a literature.^24^ AMH-derived ZnGa_2_O_4_-700 and standard ZnGa_2_O_4_ exhibit the best catalytic activities in terms of CO evolution and reaction selectivity after the calcination temperature of 700 and 850 °C,^24^ respectively (Fig. S6†). These two best catalysts for respective systems were closely examined in the following section.

**Fig. 6:**
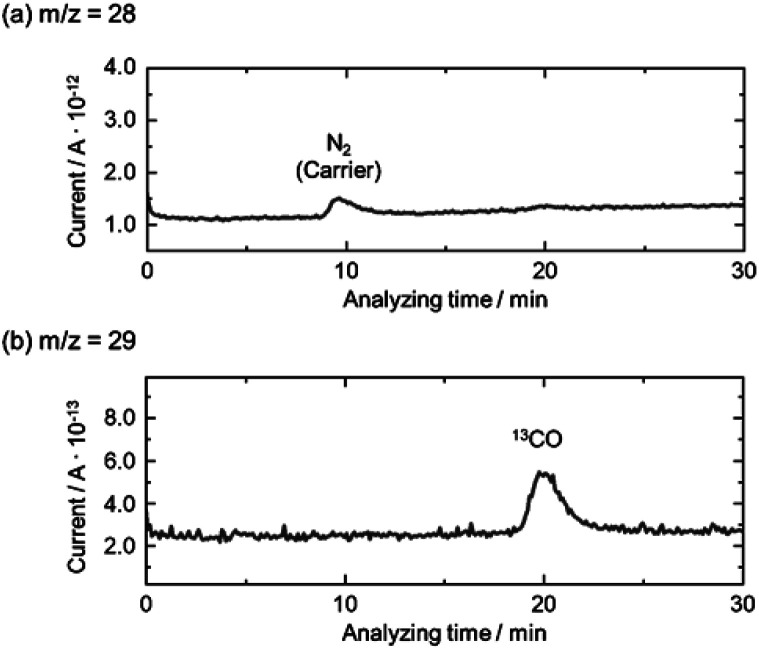
Mass spectra of (a) *m*/*z* = 28 and (b) *m*/*z* = 29 in the photocatalytic conversion of ^13^CO_2_ by H_2_O over bare AMH-derived ZnGa_2_O_4_-700 photocatalyst.

**Fig. 7:**
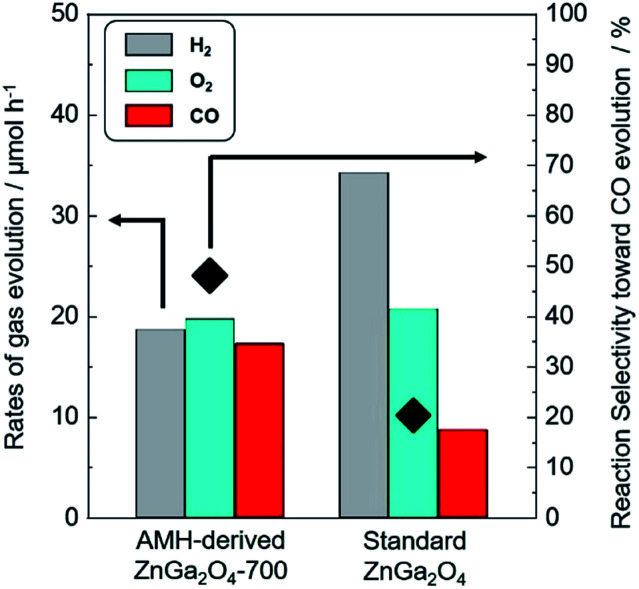
Rates of H_2_ (grey), O_2_ (sky blue) and CO (red) evolution and reaction selectivity toward CO evolution (◆) over bare AMH-derived ZnGa_2_O_4_-700 and bare standard ZnGa_2_O_4_.

The isotopic experiment was also carried out using ^13^CO_2_ as a gaseous reactant. Q-Mass spectra in *m*/*z* = 28 and *m*/*z* = 29 were collected for the photocatalytic conversion of ^13^CO_2_ with H_2_O over AMH-derived ZnGa_2_O_4_-700 (Fig. 6). The peak derived from ^13^CO is observed on Q-Mass spectrum of *m*/*z* = 29. It has been evidenced that AMH-derived ZnGa_2_O_4_-700 photocatalytically reduces introduced ^13^CO_2_ into ^13^CO. Fig. S7† shows time course of gas evolutions for photocatalytic conversion of CO_2_ with water over AMH-derived ZnGa_2_O_4_-700. The linear increase of evolved gases indicates that the present ZnGa_2_O_4_ catalyst possesses the stability in the photocatalytic reaction in aqueous solution. Furthermore, any impurities are not observed on the XRD pattern of the spent catalyst, which also supports the stability of AMH-derived ZnGa_2_O_4_-700 (Fig. S8†). Rates of gas evolution (eqn (1)–(3)) and reaction selectivity toward CO evolution (eqn (4)) over bare ZnGa_2_O_4_ catalysts (without co-catalysts) are represented in Fig. 7. Both ZnGa_2_O_4_ catalysts work as photocatalysts for CO_2_ reduction along with evolving gaseous products by reduction and oxidation in a stoichiometric ratio, (H_2_ + CO)/O_2_ = 2.0. This further supports that the formation of CO over both ZnGa_2_O_4_ catalysts is derived from not a decomposition of contaminated organic compounds but photocatalytic conversion of introduced CO_2_ molecules.^34^1
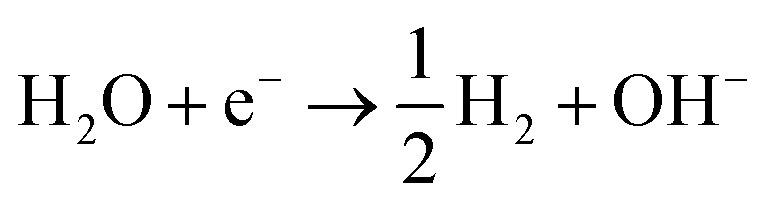

2 CO_2_ + 2H^+^ + 2e^−^ → CO + H_2_O

3
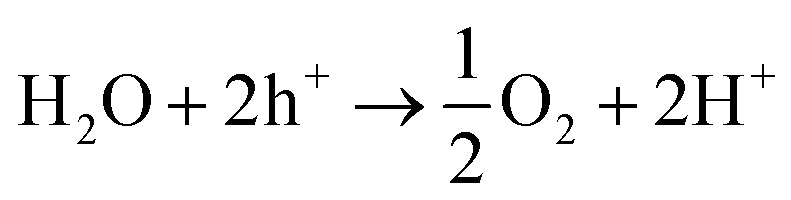
4
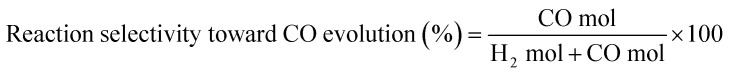


There is no difference between two photocatalysts in the formation rates of oxidation product (O_2_) and sum of reduction products (H_2_ + CO). The rates of gas evolution do not degrade during photocatalytic reaction for 5 h for both photocatalysts. It is interesting to note that the clear difference is observed in the reaction selectivity toward the CO evolution between bare AMH-derived ZnGa_2_O_4_-700 (48.0%) and bare standard ZnGa_2_O_4_ (20.3%). Energy gap (*E*_g_) estimated by UV-Vis spectroscopy, crystallite size, and specific surface area of ZnGa_2_O_4_ catalysts are summarized in Fig. S9 and Table S2.† There can be seen no relationship between these parameters and reaction selectivity toward CO evolution. More clearly, a high reaction selectivity toward CO evolution with a stoichiometric ratio, (H_2_ + CO)/O_2_ = 2.0, is observed even in the case of AMH-derived ZnGa_2_O_4_-900 (47.3%) whose *E*_g_ and *S*_BET_ are comparable to and smaller than standard ZnGa_2_O_4_, respectively. As discussed in the previous section, CO_2_ adsorption capacity per unit surface area of catalysts is larger for AMH-derived ZnGa_2_O_4_-700. The high reaction selectivity toward CO evolution over AMH-derived ZnGa_2_O_4_-700 can be reasonably explained by the high affinity for CO_2_ of AMH-derived ZnGa_2_O_4_-700.

### Photocatalytic CO_2_ reduction over Ag-loaded ZnGa_2_O_4_

**Fig. 8:**
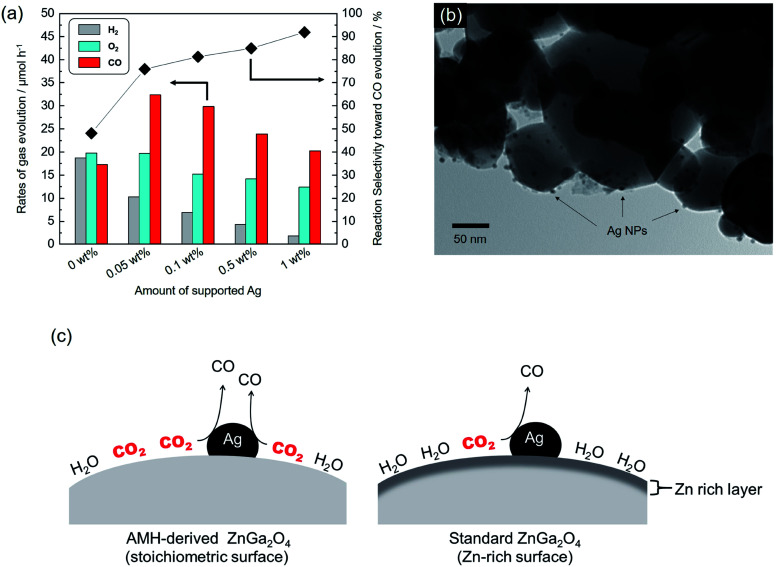
(a) Rates of gas evolution for H_2_ (grey), O_2_ (sky blue) and CO (red) and reaction selectivity toward CO evolution over Ag-loaded AMH-derived ZnGa_2_O_4_-700 at different Ag loadings. (b) TEM image of Ag-loaded AMH-derived ZnGa_2_O_4_-700 with 0.1 wt% of Ag co-catalyst. (c) Schematic illustration of photocatalytic CO_2_ reduction over AMH-derived ZnGa_2_O_4_ (left) and standard ZnGa_2_O_4_ (right) with Ag loading.

It has been well-known that metal co-catalysts, including Ag NP, are capable to reduce the recombination between excited electrons and holes.^35,36^Fig. 8(a) shows the rates of gas evolution for H_2_, O_2_ and CO, and the reaction selectivity toward CO evolution over AMH-derived ZnGa_2_O_4_-700 with Ag co-catalyst at various loadings. The rate of H_2_ evolution decreases with increasing the loaded amount of Ag co-catalyst. This trend is corresponding to those demonstrated in previous reports.^24,37,38^ Ag was reported to work as a preferential active sites for CO_2_ reduction and thereby inhibits H_2_ evolution with increasing coverage with Ag, resulting in enhanced reaction selectivity toward CO evolution. The maximum activity for CO evolution (32.3 μmol h^−1^) could be achieved at 0.05 wt% of Ag co-catalyst with a drastically improved reaction selectivity toward CO evolution (75.9%). Compared to a previous ZnGa_2_O_4_ catalyst with an optimized Ag loading of 1 wt%,^24^ 95% of Ag can be cut down in the present case, thanks to the high affinity for CO_2_ of AMH-derived ZnGa_2_O_4_-700. Fig. 8(b) shows a TEM image of AMH-derived ZnGa_2_O_4_-700 at Ag loading of 0.1 wt%. The surface of AMH-derived ZnGa_2_O_4_-700 is partially covered by dispersedly-loaded Ag NPs. Fig. 8(c) represents a schematic illustration explaining the relationship between surface properties and photocatalytic activity toward CO_2_ reduction. The surface of AMH-derived ZnGa_2_O_4_-700 with a homogeneous distribution of Zn and Ga works as an efficient CO_2_ absorber and concentrate CO_2_ to be reduced. The preferential adsorption of CO_2_ on AMH-derived ZnGa_2_O_4_-700, evidenced by CO_2_-TPD measurement (Fig. 3(b)), allows for the photocatalytic reduction of CO_2_ at catalytic active sites, vicinity of Ag co-catalysts, in a more selective manner even at a low Ag-loading.

In summary, the present work has demonstrated the formation of desirable surfaces on complex metal oxides by using precursors of interdispersed nanometric hydroxides. ZnGa_2_O_4_ was chosen as a representative system to demonstrate the present concept for yielding metal oxide photocatalysts with an enhanced catalytic activity originated from a high surface basicity. The scheme of using NPs as precursors is further expected to be available for designing a wide range of metal oxide catalysts with nano/macrotextures by integrating with the nano building block approach.^20,22,39^

## Conclusions

The metal hydroxides precursor (Zn–Ga AMH) with a large hetero-interfaces of Zn(OH)_2_ and Ga(OH)_3_ NPs was prepared through an epoxide mediated alkalization. ZnGa_2_O_4_ catalysts as a single phase were synthesized using the Zn–Ga AMH precursor. Surface analysis with Hammett indicators reveals that AMH-derived ZnGa_2_O_4_ catalysts exhibit the stronger base strength even after the calcination at high temperature, ≥700 °C, compared to ZnGa_2_O_4_ prepared through a solid phase reaction. CO_2_–TPD measurements revealed that the peak at 94 °C, originated from desorption of CO_2_, was observed only for the TPD curve of AMH-derived ZnGa_2_O_4_-700, suggesting that the surface of AMH-derived ZnGa_2_O_4_ has a higher affinity and a stronger adsorptive nature for CO_2_ than standard ZnGa_2_O_4_. Structural analysis by XPS reveals that AMH-derived ZnGa_2_O_4_-700 has a surface of the stoichiometric composition, resulting in a higher affinity for CO_2_ than standard ZnGa_2_O_4_. The bare AMH-derived ZnGa_2_O_4_-700 with a high affinity for CO_2_ exhibits the highest reaction selectivity toward the CO_2_ reduction (48.0%) among previously-reported co-catalysts-free metal oxide catalysts for the photocatalytic conversion of CO_2_ with liquid H_2_O. Additionally, the loading amount of Ag co-catalyst decreased by 95% due to the higher affinity for CO_2_. Based on these insights obtained in this representative ZnGa_2_O_4_ system, the present scheme using interdispersed hydroxide nanoparticles will pave the way to general method to tune the surface properties of metal oxide catalysts.

## Conflicts of interest

There are no conflicts to declare.

## Supplementary Material

RA-010-D0RA00710B-s001
