# Supplementary material for: Imparting CO2 reduction selectivity to ZnGa2O4 photocatalysts by crystallization from hetero nano assembly of amorphous-like metal hydroxides
Source: RSC Adv. 2020 Feb 25;10(14):8066–73. doi: 10.1039/d0ra00710b (PMC9049919; doi:10.1039/d0ra00710b)
Supplement: RA-010-D0RA00710B-s001 [file RA-010-D0RA00710B-s001.pdf]

Electronic Supplementary Material (ESI) for RSC Advances.
This journal is © The Royal Society of Chemistry 2020

Supporting Information

Imparting CO_2 reduction selectivity to $ZnGa_2O_4$ photocatalysts by crystallization from hetero nano assembly of amorphous-like metal hydroxides.

Masanori Takemoto, Yasuaki Tokudome, Soichi Kikkawa, Kentaro Teramura*, Tsunehiro Tanaka, Hidenobu Murata, Atsushi Nakahira, Kenji Okada and Masahide Takahashi*

*corresponding authors

**Experimental**	**2**
**Characterization**	**3**
**Figure S1**	**4**
**Figure S2**	**5**
**Figure S3**	**6**
**Figure S4**	**7**
**Figure S5**	**8**
**Figure S6**	**9**
**Figure S7**	**10**
**Figure S8**	**11**
**Figure S9**	**12**
**Table S1**	**13**
**Table S2**	**13**
**Reference**	**14**

Experimental

Solid state reaction

The synthesis was performed according to a previous-reported $ZnGa_2O_4$ synthesis.[1] ZnO (0.65 g), Ga_2O_3 (1.50 g) and deionized water (2.5 mL) were mixed in a mortar for 30 min to yield homogeneous slurry. The slurry was dried in oven at 80 °C for 15 min. 1 mL of deionized water was added to the dried powder and the slurry was mixed for 30 min. Then, the slurry was dried in oven at 80 °C for 1 h, and calcined at 700 °C or 850 °C for 12 h.

Characterization

Electric conductivity measurement

The total amount of ionic species (Zn^{2+} + Ga^{3+}) consumed to yield metal hydroxide through the alkalization by epoxide ring-opening was calculated from an electric conductivity measurement according to the previous report. [2] The solution at a molar ratio of $Zn(NO_3)_2 \cdot 6H_2O$: $Ga(NO_3)_3 \cdot nH_2O$: ultra-pure water: ethanol: PO = 1: 2: 134: 66: 45 was measured. The data was collected at 24 h after the addition of PO to the precursor solution.

Calculation of crystalline size of $ZnGa_2O_4$ catalysts

Crystallite size of $ZnGa_2O_4$ was calculated using Scherrer's equation by reference to the previous reports,[3,4] $D = K\lambda/(B\cos\theta)$. Here, K is a constant (0.9), λ is the wavelength of CuKα radiation (0.154 nm), B is the Full Width of Half Maximum (FWHM) of the diffraction peak centered at 35.73 °, and θ is Bragg's angle.

X-ray photoelectron spectroscopy (XPS) of $ZnGa_2O_4$ catalysts

Powderly sample was molded into a pellet (φ = 7 mm). The pellet was mounted on the sample holder by using conductive carbon tape. All the binding energies were refereced to the C 1s peak at 284.8 eV of the surface adventitious carbon. The surface atomic ratio (Zn/Ga) was calculated from the integrated intensity of Zn $2p_{3/2}$ and Ga $2p_{3/2}$ spectra by using corrsponding relative sensitivity factors.

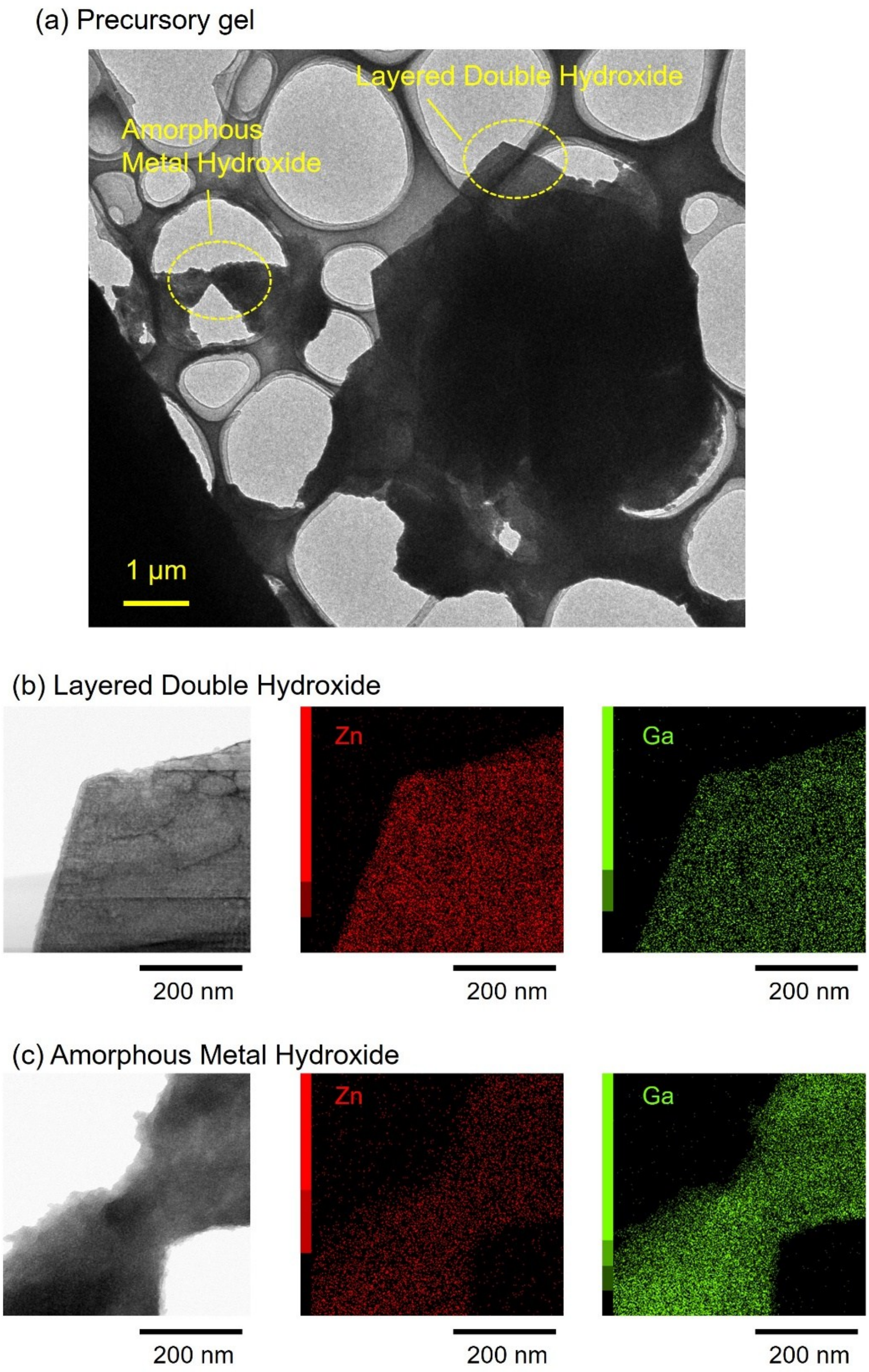

Figure S1 (a) FE-TEM image of precursory wet gel obtained from an aqueous solution of $Zn(NO_3)_2{\cdot}6H_2O$ and $Ga(NO_3)_3{\cdot}nH_2O$ under a highly supersaturated condition induced by epoxide-mediated alkalization. Enlarged views and EDS mappings of Zn and Ga of (b) Layered Double hydroxide and (c) Amorphous Metal Hydroxide as shown in Fig. S1(a).

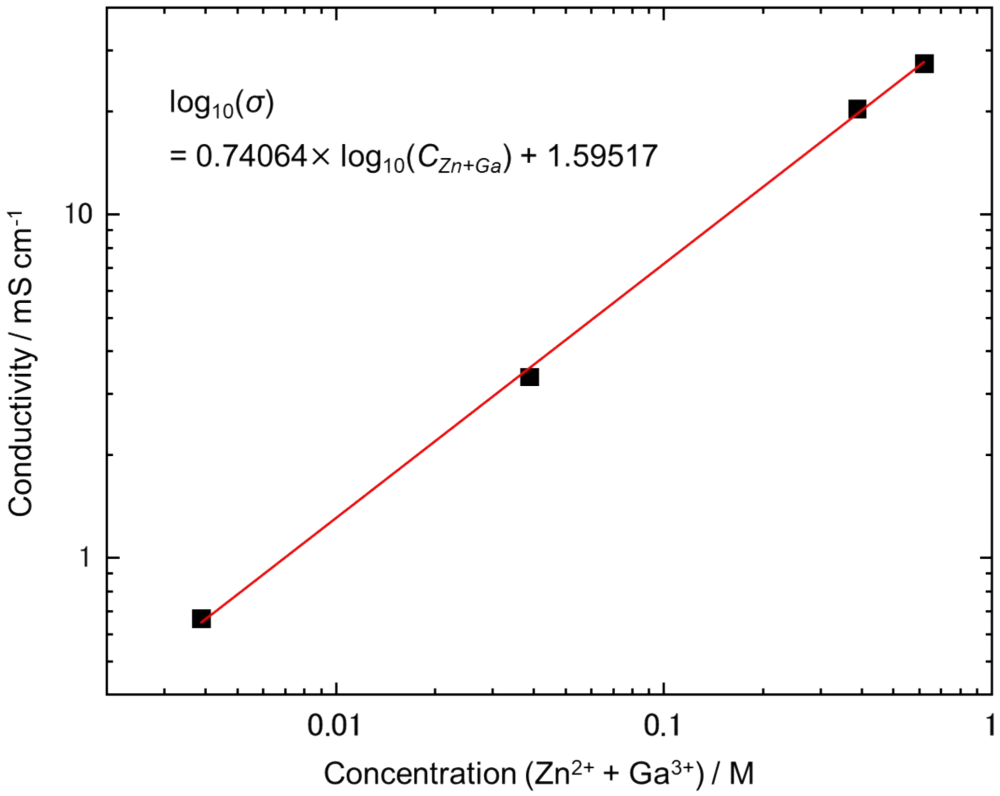

Figure S2 Calibration curve for determining the amount of comsumed ionic species druing the precipitation of hydroxides. The calibration is $\log_{10}(\sigma) = 0.74064 \times \log_{10}(C_{Zn+Ga}) + 1.59517$, where σ and C_{Zn+Ga} are the conductivity of the solution and the total concentration of metal ions (Zn^{2+} + Ga^{3+}), respectively. The value of conductivity of the reacting solution at 24 h is 2.42 mS cm^{-1}, corresponding to 0.0231 M, where 96.3% of ionic species are consumed to precipitate hydroxides.

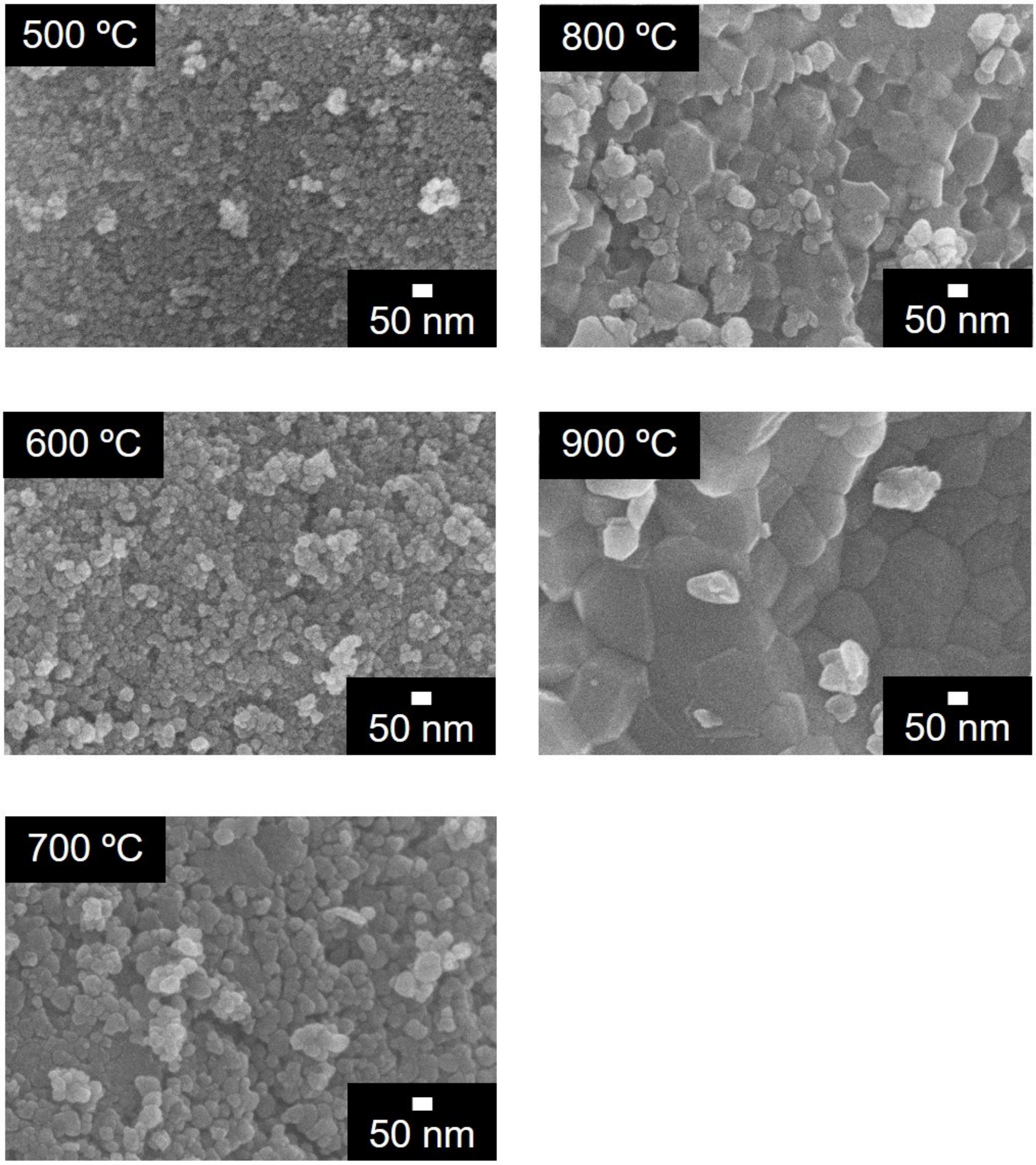

Figure S3 SEM images of AMH-derived $ZnGa_2O_4$ catalysts.

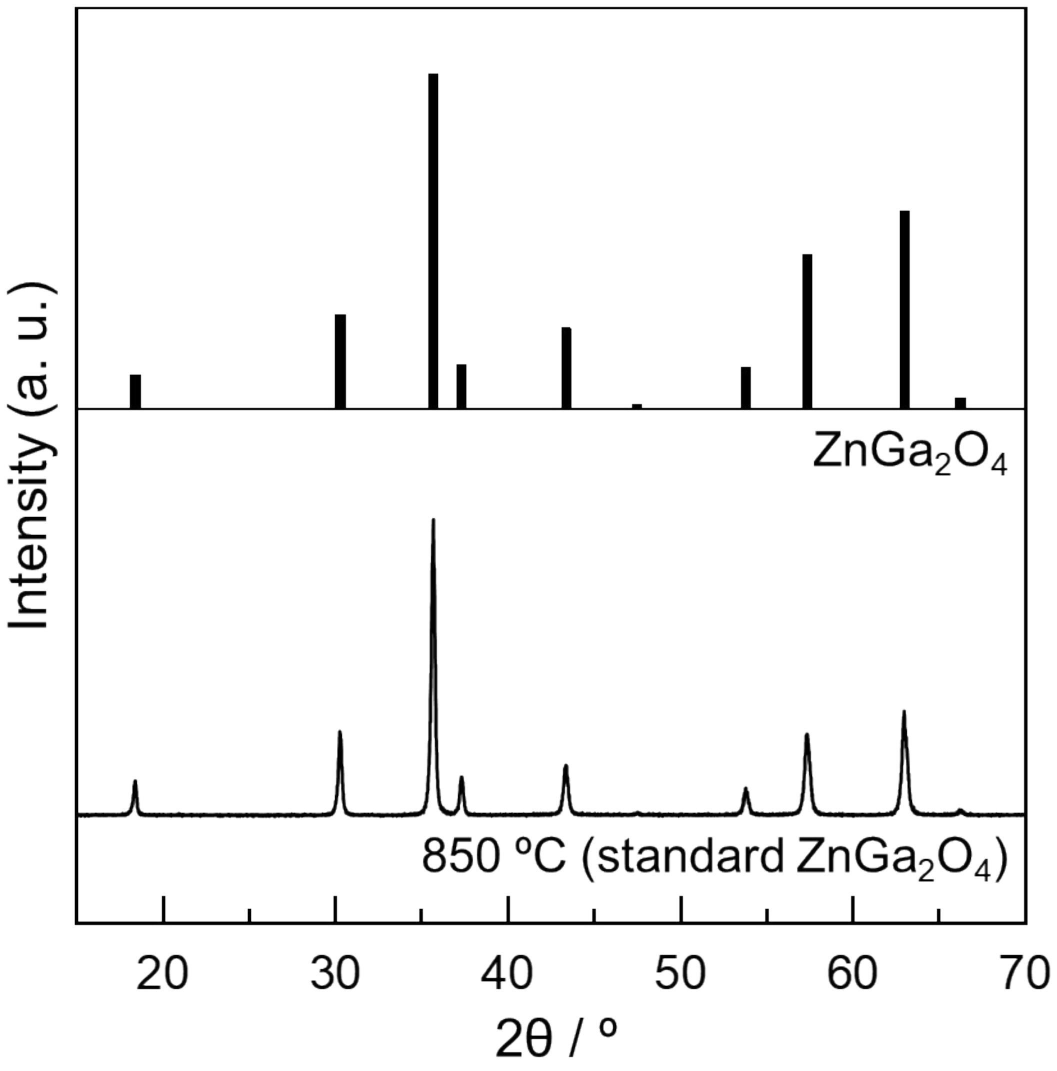

Figure S4 PXRD patterns of $ZnGa_2O_4$ prepared from a mixture of ZnO and Ga_2O_3 powders by calcination at 850 °C. $ZnGa_2O_4$ obtained as a single phase was named as "standard $ZnGa_2O_4$". Crystallite size and BET specific surface area of standard $ZnGa_2O_4$ are 29 nm and 1.3 $m^2\ g^{-1}$, respectively.

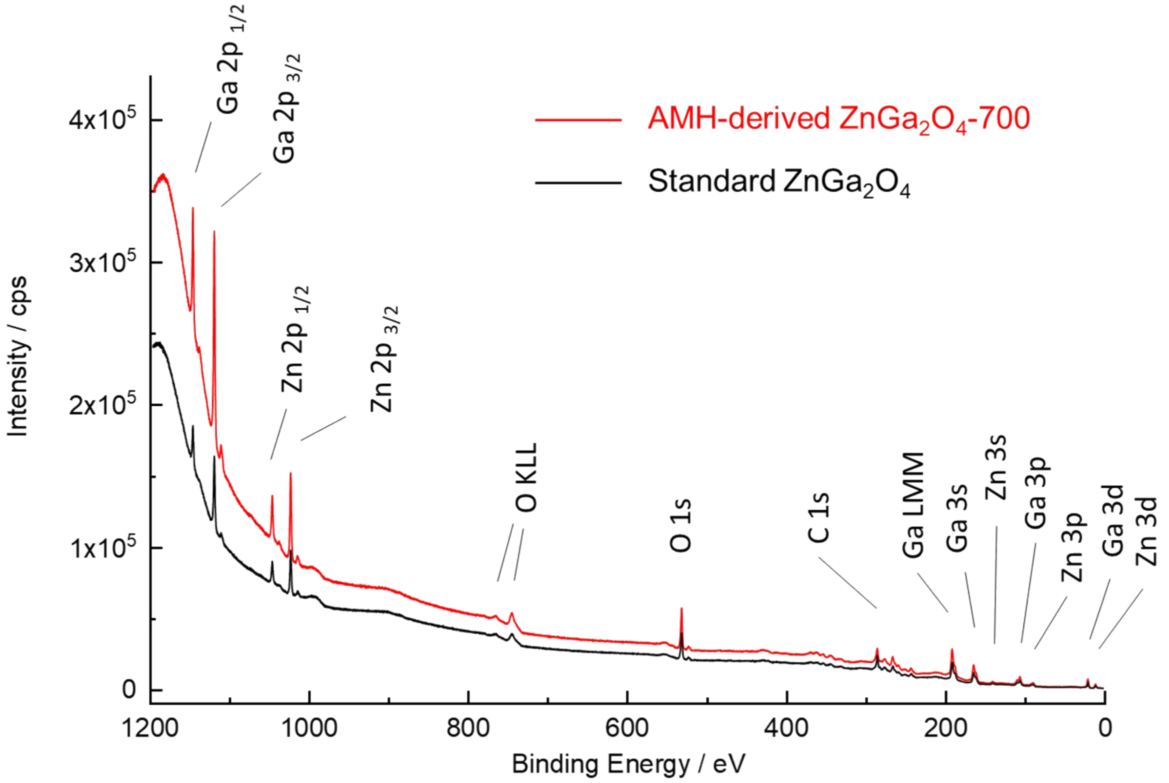

Figure S5 Wide scan XPS spectra of AMH-derived $ZnGa_2O_4$-700 (red line) and standard $ZnGa_2O_4$ (black line).

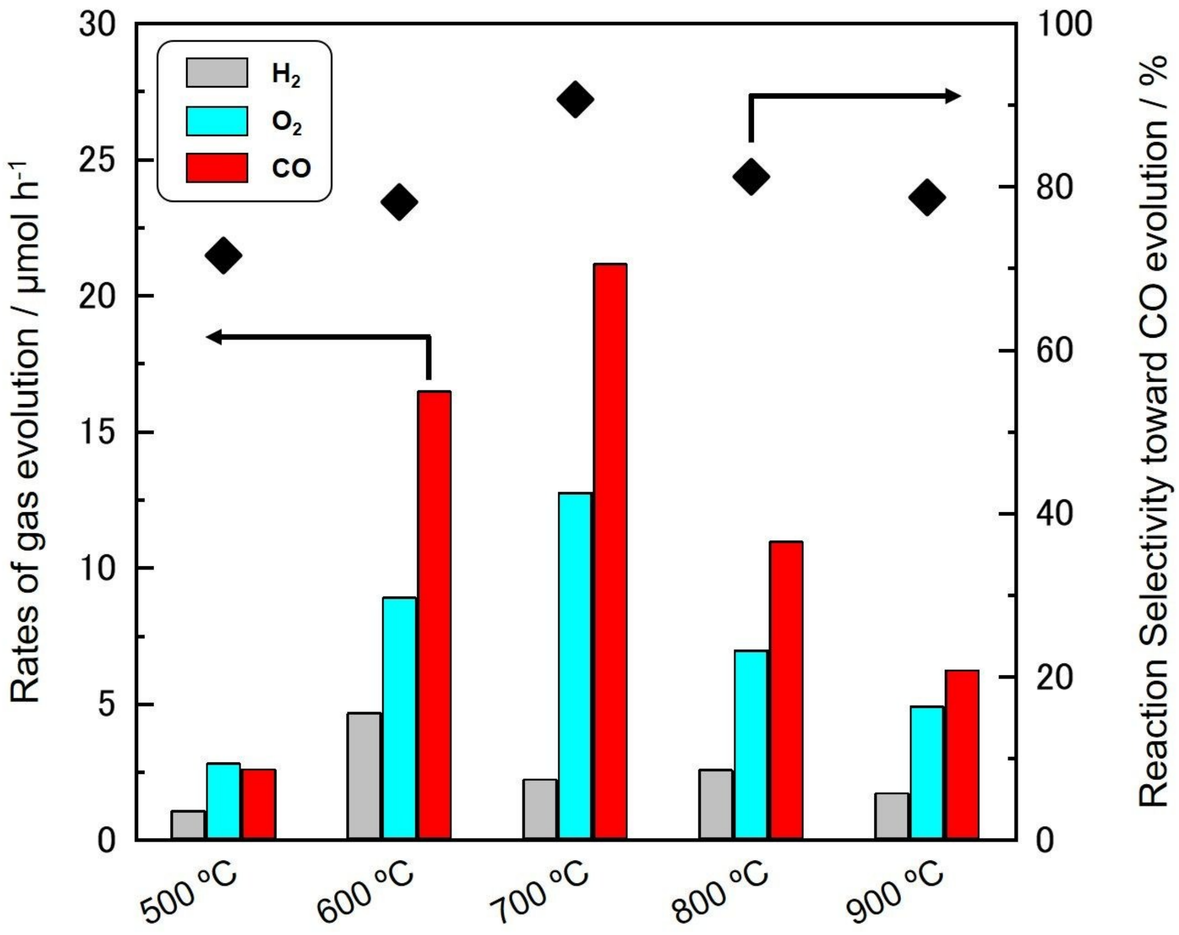

Figure S6 Rates of H_2 (grey), O_2 (sky blue), and CO (red) evolution and reaction selectivity toward CO evolution (◆) over Ag-loaded AMH-derived $ZnGa_2O_4$ prepared by calcination at various temperatures for 12 h; the loading amount of Ag co-catalyst: 1.0wt%.

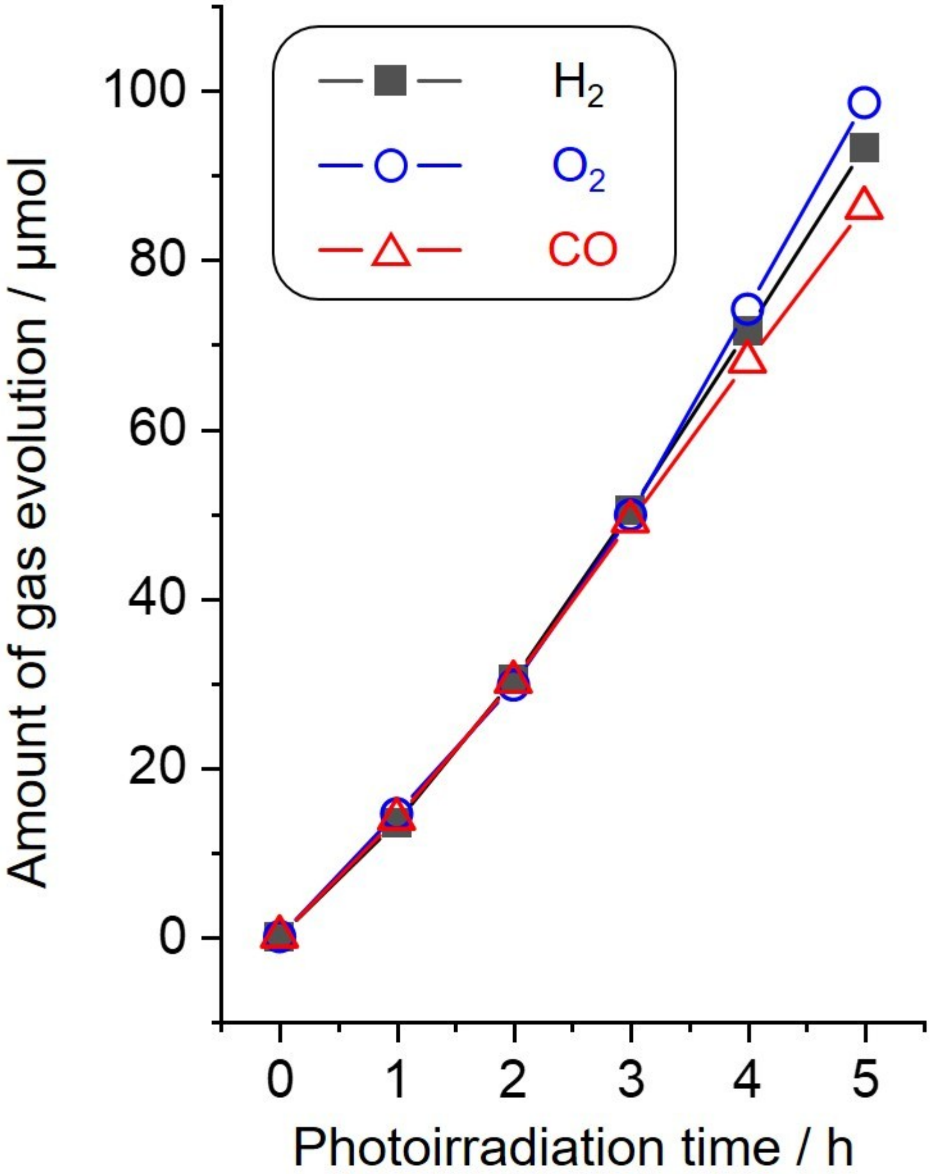

Figure S7 Time course of gas evolutions for photocatalytic conversion of CO_2 with H_2O over bare AMH-derived $ZnGa_2O_4$-700.

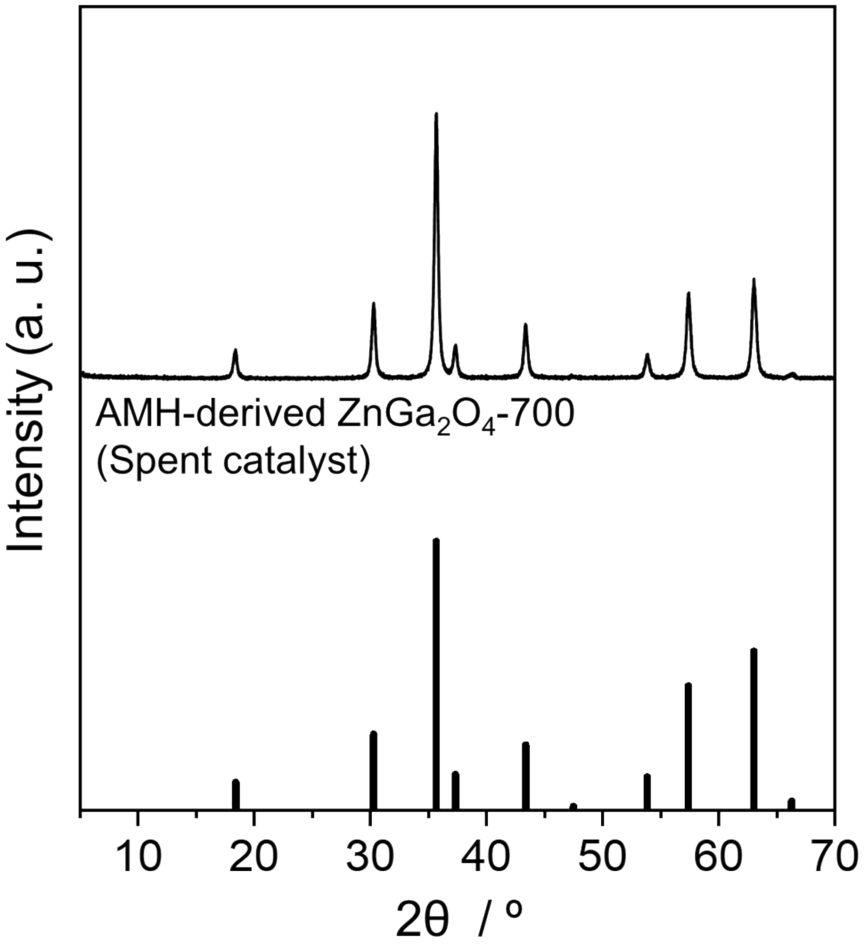

Figure S8 PXRD pattern of AMH-derived $ZnGa_2O_4$-700 after the photocatalytic CO_2 reduction in water.

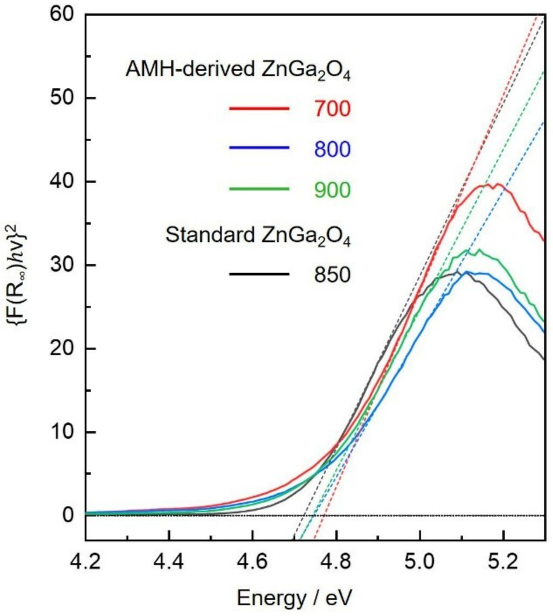

catalyst	Temperature / °C	E_g / eV
AMH-derived $ZnGa_2O_4$	700	4.8
	800	4.7
	900	4.7
Standard $ZnGa_2O_4$	850	4.7

Figure S9 UV-Vis spectra and estimated E_g values of $ZnGa_2O_4$ catalysts (without Ag co-catalyst). There can be seen no dependence of the optical absorption property of $ZnGa_2O_4$ catalysts (E_g: 4.6 - 4.8 eV) on the resultant catalytic activities. Catalysts after photocatalytic reaction were collected and measured.

Table S1 Base strength, crystallite size and specific surface area of bare AMH-derived $ZnGa_2O_4$ and standard $ZnGa_2O_4$.

Photocatalyst	Precursor	Temperature [ºC]	Crystallite size [nm]	Specific surface area [$m^2\ g^{-1}$]	Base strength
AMH-derived $ZnGa_2O_4$	Zn-Ga AMH	500	6.9	26	$15 < H\_$
		600	13	15	$15 < H\_$
		700	20	5.0	$9.8 < H\_ < 15$
		800	25	4.7	$9.8 < H\_ < 15$
		900	39	0.53	$9.8 < H\_ < 15$
Standard $ZnGa_2O_4$	ZnO + Ga_2O_3	850	29	1.3	$H\_ < 7.2$

Table S2 Summary of crystallite size, specific surface area and reaction selectivity toward CO evolution in photocatalytic conversion of CO_2 with H_2O of bare $ZnGa_2O_4$ catalysts.

Photocatalyst	Temperature [ºC]	Crystallite size [nm]	Specific surface area [$m^2\ g^{-1}$]	Reaction selectivity toward CO evolution [%]
AMH-derived $ZnGa_2O_4$	700	20	5.0	48.0
	800	25	4.7	40.3
	900	39	0.53	47.3
Standard $ZnGa_2O_4$	850	29	1.3	20.3

Reference

1. Z. Wang, K. Teramura, S. Hosokawa and T. Tanaka, *J. Mater. Chem. A*, 2015, **3**, 11313-11319.
2. N. Tarutani, Y. Tokudome, M. Jobbágy, F. A. Viva, G. J. A. A. Soler-Illia and T. Masahide, *Chem. Mater.*, 2016, **28**, 5606-5610.
3. J. S. Kim, J. S. Kim and H. L. Park, *Solid State Commun.*, 2004, **131**, 735-738.
4. Q. Shi, J. Zhang, C. Cai, L. Cong and T. Wang, *Mater. Sci. Eng.: B*, 2008, **149**, 82-86.
